# The Prophylaxis of Erysipelas

**Published:** 1882-05

**Authors:** Alfred Stillé


### THE PROPHYLAXIS OF ERYSIPELAS.

1. The utmost purity of the air should be preserved in all apartments used habitually by day, or for sleeping, and especially in hospital wards and other places occupied by the sick.

2. All patients suffering from erysipelas should be isolated, and nothing that has been used by or for them, and least of all surgical instruments, should be employed for non-erysipelatous patients. On the same principle, in climates and seasons which make it possible to treat the wounded in tents or temporary wooden hospitals, such as were used during our civil war, the danger of erysipelas is reduced to a minimum by so doing.

3. On no account should a puerperal patient be confined in a house infected with erysipelas, nor attended by any physician who has recently had charge of an erysipelatous case.

4. A surgical ward should never be in close proximity to a lying-in ward, nor even in the same building, and the attendants in one should hold no communication with those of the other.

5. During general or local epidemics of erysipelas, all cutting operations should be, if possible, avoided, it being remembered that the danger of erysipelatous infection of wounds is in direct proportion to their extent.

6. For these reasons, it is held by some surgeons that subcutaneous incisions should, under such circumstances, be preferred, and that the surface of recent wounds should be protected by the nitrate of silver film. —*Prof. Alfred Stillé, in International Encyclopedia of Surgery.*

